# The Penile Microbiota in Uncircumcised and Circumcised Men: Relationships With HIV and Human Papillomavirus Infections and Cervicovaginal Microbiota

**DOI:** 10.3389/fmed.2020.00383

**Published:** 2020-07-30

**Authors:** Harris Onywera, Anna-Lise Williamson, Julia Ponomarenko, Tracy L. Meiring

**Affiliations:** ^1^Institute of Infectious Disease and Molecular Medicine, University of Cape Town, Cape Town, South Africa; ^2^Division of Medical Virology, Department of Pathology, Faculty of Health Sciences, University of Cape Town, Cape Town, South Africa; ^3^Center for Genomic Regulation (CRG), The Barcelona Institute of Science and Technology, Barcelona, Spain; ^4^SAMRC Gynaecological Cancer Research Centre, University of Cape Town, Cape Town, South Africa; ^5^University of Pompeu Fabra, Barcelona, Spain

**Keywords:** penile microbiota, male circumcision, human immunodeficiency virus (HIV), human papillomavirus (HPV), bacterial vaginosis (BV), cervicovaginal microbiota

## Abstract

While the human microbiota especially that of the gut, cervix, and vagina continue to receive great attention, very little is currently known about the penile (glans, coronal sulcus, foreskin, and shaft) microbiota. The best evidences to date for the potential role of the penile microbiota in human immunodeficiency virus (HIV) and other sexually transmitted infections (STIs) acquisition have come from studies examining medical male circumcision. We are still at the foothills of identifying specific penile bacteria that could be associated with increased risk of STI/HIV acquisition. In this review, we summarize the available literature on the human penile microbiota and how it is impacted by circumcision. We also discuss the potential role of penile microbiota in STIs and its impact on cervicovaginal microbiota. Taken together, the findings from the penile microbiota studies coupled with observational studies on the effect of male circumcision for reduction of STI/HIV infection risk suggest that specific penile anaerobic bacteria such as *Prevotella* spp. potentially have a mechanistic role that increases the risk of genital infections and syndromes, including bacterial vaginosis in sexual partners. Although penile *Corynebacterium* and *Staphylococcus* have been associated with healthy cervicovaginal microbiota and have been found to increase following male circumcision, further investigations are warranted to ascertain the exact roles of these bacteria in the reproductive health of men and women. This review aims to address existing gaps and challenges and future prospects in the penile microbiota research. The information described here may have translational significance, thereby improving reproductive health and management of STI/HIV.

## Introduction

Although it is well-documented that the human body is uniquely inhabited by site-specific microbiota (1, 2), information on the penile (glans, coronal sulcus, foreskin, and shaft) microbiota remains remarkably deficient. Initial investigation of the penile bacteria relied on classical approaches such as culture (3, 4). A caveat to the culture method is that about 99% of microorganisms discovered to date are yet to be cultured (5). The development of next generation sequencing (NGS) technologies utilizing marker genes, such as the bacterial 16S ribosomal rRNA (rRNA) gene, have allowed us to begin understanding the composition, diversity, stability, and function of the penile microbiota.

The recent interest in penile microbiota has been sparked by the findings from randomized control trials (RCTs) of medical male circumcision (MMC, posthectomy) for risk reduction of human immunodeficiency virus (HIV) (6–9), high-risk human papillomavirus (HPV) infection (10), and female sexual partners' vaginal infections and syndromes (11, 12). Despite the beneficial effects of male circumcision in reducing the risk of STI/HIV, vaginal infections and other syndromes as observed in these RCTs (6–11) and analogous studies (13–19), the biological reasons are not entirely understood (20). Some of the proposed biological mechanisms responsible for the protective effect of male circumcision on sexually transmitted infections (STIs) transmission, particularly HIV and HPV infections, include (i) reduction in local immune inflammation in the penile tissues, which prevents loss of epithelial barrier integrity (20) and reduces the density of HIV-susceptible cells (20, 21), as well as (ii) keratinization of the glans, although this is a less likely mechanism (20, 22–24). It has been suggested that penile inflammation may be induced by penile microbiota (20, 25). Thus, alteration of penile microbiota especially through male circumcision may have profound benefits in reducing the risk for STI/HIV.

In this review, we first present an overview of the penile microenvironment and define studies on male circumcision that led to the view that the penile microbiota could have an impact on genital infections and syndromes. We also revisit the available literature on the penile microbiota and present data suggesting the interaction of penile microbiota with cervicovaginal and urethral/urine microbiota. Finally, we discuss the current developments, gaps, and future prospects on penile microbiota.

### The Anatomy of the Human Penis—Defining the Penile Microenvironment in Uncircumcised and Circumcised Penis

**Figure 1:**
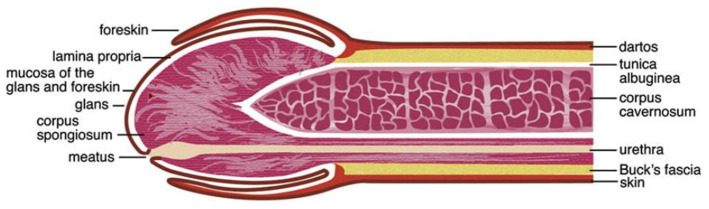
Anatomy of the human penis. The figure is taken from the review entitled “Benign Diseases and Neoplasms of the Penis” by Wasco and Shah (26), with permission to reproduce it.

The human penis consists of the glans, corona (junction between the glans and penile shaft), urethra, corpora cavernosa, corpus spongiosum, and prepuce (foreskin in uncircumcised men; Figure 1) (26–29). The various surfaces of the penis represent different microenvironments, which differ in properties such as oxygen availability, keratinization and wetness, which provide suitable niches for different bacterial communities.

The external surface of a relaxed penis, including the outer foreskin, is covered with “dry” keratinized squamous epithelial layer that is relatively impermeable to STIs in the absence of epithelial trauma and/or lesions (27–29). In the majority of uncircumcised men, the prepuce often covers the glans, corona, and the meatus (tip of the penis) (27, 29, 30). The prepuce has the following layers: mucosa (mucosal epithelium/inner plate of the prepuce), lamina propria, dartos muscle, dermis, and glabrous outer epithelium (29). The inner foreskin is covered by a keratinized squamous epithelium that resembles that of the mucosa of the oral cavity, esophagus, and vagina (30). It is still debated whether the inner and outer foreskin keratin layers differ in their thickness (24, 31–33).

The prepuce contains ectopic sebaceous glands that produce smegma, whose contents are prostatic and seminal secretions, desquamated epithelial cells, and mucus from urethral glands of Littré (23). Studies investigating the topographical and temporal diversity of the human skin microbiota have reported that the skin microbiota is impacted by the density of sebaceous glands, with high-density sites favoring growth of lipophiles, e.g., *Propionibacterium acnes* (2). A study conducted among 40 South Korean boys aged 3 months to 9 years and 11 months found that the smegma was colonized by bacteria, including uropathogens (34). Comparison of the prevalence of the most frequently bacteria isolated from glans with vs. without smegma showed a wide variation, differing by 1.6–5.7 times (*Escherichia coli*: 27.3 vs. 4.8%, *Enterococcus avium*: 22.7 vs. 9.5%, and *Enterococcus faecalis*: 18.2 vs. 28.6%) (34). Secretions from the urethral glands of Littré, prostate, and seminal vesicle are said to keep the preputial sac and glans moist (23, 29). The preputial sac may also be moistened by a fluid transudate from the rich vascular plexus of the prepuce mucosa (29). The subpreputial epithelium that covers the glans, corona, and inner surface of the prepuce is made up of mucosal (“wet”) squamous epithelial cells (27–30, 35). The length of the prepuce of uncircumcised men has been associated with subpreputial wetness (36). This subpreputial penile wetness and large preputial surface area have been associated with HIV infection (37, 38).

Circumcision removes most, if not all of the preputial skin and its mucosa, thereby leaving the glans exposed (27, 30). It has been reported that exposure of the glans by circumcision causes it to undergo keratinization (39). However, histological observations of cadaveric penile tissues found that the glans epithelia of six uncircumcised and seven circumcised men were equally keratinized (22). Thus, debate remains concerning whether the glans keratinizes post-circumcision. Circumcision also leads to the elimination of the moist anoxic microenvironment of the subpreputial space (40). The meatus, urethral orifice, and the penile skin, including the anoxic subpreputial space, are suitable niches for microbes (27, 28, 40–42) and at times pathogens (3, 4). The mucosal epithelial of the penis has immunological cells that act against or promote infections by pathogens (21, 31, 35, 43). Physical and immunological alterations affecting the penis are therefore likely to influence its colonization with microbes (40, 42).

### Male Circumcision and Impact on Sexually Transmitted Infections

There are two types of male circumcision: medical and traditional circumcision. MMC involves the surgical removal of the entire foreskin by a medical practitioner (44, 45). On the other hand, traditional male circumcision (TMC) is usually performed by a medically untrained provider in a non-clinical setting, with either the foreskin partially or fully (completely) removed (46–50) using different cutting techniques (51). TMC is common in many settings within sub-Saharan Africa (45–53), and is performed as a ritual to initiate the males into manhood (46, 49).

#### Medical Male Circumcision as an STI Prevention Strategy

Removal of the prepuce by MMC significantly reduces the risk of STIs, including herpes simplex virus type 2 (HSV-2), syphilis, gonorrhea, HPV, and HIV (6–8, 12, 14, 15, 17–19, 54). Three RCTs, conducted in South Africa (6), Kenya (7), and Uganda (8) observed that MMC reduced heterosexual HIV transmission in men without behavioral disinhibition (risk compensation behavior) by about 60%. Two recent systematic reviews and meta-analyses (55, 56) found that male circumcision reduces prevalent HPV by between 32 and 43%. The foreskin has been reported to have a higher prevalence of HPV compared to other penile sites in uncircumcised men (56). Furthermore, additional studies have noted that the glans/corona of an uncircumcised penis has a higher prevalence (18) and reduced clearance rates of HPV compared to that of a circumcised penis (17). The protective effect of male circumcision against HPV infection has been found to be more enhanced in glans/corona and urethra relative to sites more distal to the prepuce, such as the penile shaft and scrotum (56). Circumcision is claimed to cause the glans to thicken (39), possibly becoming more resistant to microabrasions and less susceptible to pathogens such as HPV (14). Circumcision has been associated with maintaining good penile hygiene, which may potentially reduce the risk of STIs (14). As mentioned earlier, circumcision also eliminates the moist microenvironment under the foreskin, which may favor colonization by pathogens (25, 40, 57). Initially, there was no clear evidence that male circumcision reduces the risk of STIs, including HIV (58); but following the observed considerable protective effects of MMC against HIV/STIs (9, 55, 56), male circumcision has been cited as an essential and effective element of HIV/STIs prevention strategies not only in Africa, but the rest of the world as well (59). Furthermore, there is evidence that male circumcision may also be protective against multiple STIs in sexual partners (11, 12).

MMC may also reduce the risk of urinary tract infections (UTIs) in circumcised men. This view is based on epidemiological studies from medical records of male infants that found a 10–20-fold greater incidence of UTIs in uncircumcised infants compared to circumcised infants (60, 61). Although not demonstrated, one of the epidemiological studies believed that the increased incidence of UTIs in an uncircumcised infant could have been partly caused by an increased interaction between the urethra and fecal bacteria (60). A meta-analysis of nine published studies on infancy circumcision status and risk of UTIs found a 5–89-fold increased risk of UTIs in uncircumcised males (61). One might argue that these findings may not necessarily be extrapolated beyond the infant subpopulation. However, a systematic review and meta-analysis by Morris and Wiswell (62) that included male infants, adolescents and adults, found that circumcision reduced lifetime risk of UTIs.

#### Traditional Male Circumcision as an STI Prevention Strategy

TMC may offer some level of protection against HIV infection (16, 52). For example, a cross-sectional comparative study based on 18 demographic and health surveys conducted in sub-Saharan Africa (16), where TMC is predominant, strongly associated circumcision status with reduced risk of HIV infection. However, a population-based survey of predominantly traditionally circumcised sexually active South African men found no association between circumcision status and HIV infection (47). A more recent study investigating the association between the type of male circumcision (medical vs. traditional) and HIV status on a Basotho cohort noted that traditionally circumcised men were more likely to be HIV-infected than medically circumcised men (45).

The differences in the protective effect of TMC on HIV infection are presumably due to variations in the age of coitarche (sexual debut) and amount of foreskin removed during circumcision (45–47, 50, 51, 54). A study that assessed the variations in TMC practices and their association with HIV status among South African men observed that partially circumcised and uncircumcised men had the same risk for HIV infection, which was significantly greater than that of fully circumcised men (63). Differences in HIV risk between medically and traditionally circumcised men may be attributable to lack of HIV risk reduction counseling or formal counseling received in traditionally circumcised men (64, 65), higher rates of complications (infections and delayed wound healing) among traditionally circumcised men compared to medically circumcised men (6–9, 46, 49–51), time of resumption of sexual activity during the post-circumcision wound healing-healing period (66), and inaccurate self-reporting of male circumcision status (48, 67) [an error which may be more pronounced if the information is obtained from the female sexual partners (67)] and foreskin status (amount of foreskin that covers the glans in a non-erectile condition) (68) according to the classification by Kayaba et al. (69). Partial removal of the foreskin may still maintain subpreputial penile wetness akin to that of uncircumcised men. This subpreputial penile wetness has been associated with HIV infection (37). This penile wetness could be acting as a proinflammatory mucosal immune milieu, which is also enriched with highly susceptible HIV target cells (43).

### An Update on the Human Penile Microbiota: 2020

The penile (glans, coronal sulcus, and shaft) microbiota remains largely understudied. To date, there are only ten published papers that have used NGS technologies to examine this topic. Relevant published studies included in this review's section were extracted from PubMed and Google Scholar. Key search words included “penis,” “penile,” “penile skin,” “penile shaft,” foreskin,” “prepuce,” “preputial,” “subpreputial,” “glans,” “corona,” “coronal sulcus,” “microbiota,” “microbiome,” “bacterial communities,” “circumcision,” “circumcised,” uncircumcised,” “human,” “male,” and “men.” These multiple keywords used for literature search were used in combination. Literature search was restricted to articles that described original research studies on penile microbiota and were published only in English language. The findings of these publications are summarized in Table 1, ranked by the recency of the publication. Of these studies, five were conducted on a Ugandan cohort (25, 40, 42, 57, 72), two on a U.S. cohort (41, 73), and one each on a South African (53), an Australian (71), and a Spanish cohort (70). Two of these studies focused on changes in microbiota following MMC (40, 57); whereas the rest examined the penile microbiota of uncircumcised and/or medically or traditionally circumcised men (25, 41, 53, 71–73). The ten studies characterized the penile microbiota using swabs from either glans alone (70), coronal sulcus alone (25, 40, 42, 57, 72), coronal sulcus and glans (71), or glans, coronal sulcus, and penile shaft (hereafter referred as penile skin) (41, 53). These studies targeted the penile microbiota using the hypervariable V1–V3, V3–V5 (73), V3–V4 (25, 40, 53, 70, 71), V3–V6 (42, 57, 72), V4–V6 (41), and V6–V9 regions of the 16S rRNA gene (73).

**Table 1:** Summary of findings from penile microbiota studies carried out to date.

**Study description/aim**	**Number of participants and study design**	**Penile microenvironment and region of 16S rRNA targeted (NGS technology)**	**Study cohort**	**Main finding(s)**	**Predominant bacterial taxa in cross-sectional penile specimens**	**Investigators (year of publication)**
To investigate the penile microbiota of HPV-infected men as well as the impact of HIV status	238 (including 88 HIV-seropositive, 130 HPV-positive, 102 HR-HPV-positive, and 215 circumcised) men in a heterosexual relationship	• Penile shaft, foreskin (if uncircumcised) and glans • V3–V4 hypervariable region (Illumina MiSeq)	South African	• Penile microbiota clustered into 6 CSTs. CST-1, was the most prevalent (53.4%) and was dominated by *Corynebacterium*. The most dominant genera (in decreasing relative abundances) in the other CSTs were *Corynebacterium*, unclassified *Clostridiales*, and *Porphyromonas* in CST-2 (prevalence: 9.2%); *Gardnerella* and *Corynebacterium* in CST-3 (8.8%); *Chryseobacterium, Corynebacterium*, and *Acinetobacter* in CST-4 (7.6%); and *Prevotella*, unclassified *Clostridiales, Corynebacterium*, and *Porphyromonas* in CST-5 (18.5%). CST-6 (2.5%) was dominated by *Lactobacillus*, with very low relative abundance of *Corynebacterium*. • Men in CST-1 had fewer HR-HPV infections compared to men in CSTs 2–6. Specifically, men in CST-5 were significantly more likely to have HPV or HR-HPV infections compared to men in CST-1. • Men with HR-HPV infections had greater relative abundances of the anaerobic BV-associated bacteria (*Prevotella, Peptinophilus*, and *Dialister*) and lower relative abundance of *Corynebacterium* compared to men without HR-HPV infection. • HIV did not impact CST. However, the penile microbiota of HIV-infected men was associated with greater relative abundance of *Staphylococcus*. • Families *Veillonellaceae, Prevotellaceae, Porphyromonadaceae*, unclassified *Clostridiales*, and *Clostridiales Incertae Sedis XI* positively correlated with one another whereas they negatively correlated with *Corynebacteriaceae, Moraxellaceae, Flavobacteriaceae, Pseudomonadaceae, Oxalobacteraceae, Staphylococcaceae, Bifidobacteriaceae*, and *Lactobacillaceae*, although *Clostridiales Incertae Sedis XI* was positively correlated with *Corynebacteriaceae*.	• *Corynebacterium* and *Prevotella* were found to be the most abundant genera.	Onywera et al. (53)
To examine the effect of oral and vaginal sex over the microbiota of heterosexual couple who reported recurrent vaginal and oral infections after sexual intercourse	A case report of one heterosexual couple (that included an uncircumcised male partner) in monogamous sexual relationship	• Glans • V3–V4 hypervariable region (Illumina MiSeq)	Spanish	• Condomless sexual contact significantly reduced the relative abundance of *Lactobacillus* in the vagina and caused vaginal dysbiosis that lasted at least for 1 week. • Relative abundance of *Lactobacillus* in the penis remarkably increased following oral and condomless vaginal sex. • Relative abundance of penile *Corynebacterium* increased after condomless sexual relationship. • Vaginal and penile microbiota were relatively stable over time (in the absence of sexual activity). • Several genital genera were significantly affected after 15 days of intermittent H_2_O_2_ washes (penile washes and intravaginal irrigations). The proportion of vaginal *Lactobacillus* decreased concurrently with increase in the proportions vaginal *Prevotella* and *Atopobium*. Penile washes eliminated *Prevotella*, increased the proportion of penile *Klebsiella* and decreased *Mycobacterium, Finegoldia*, and *Ralstonia*.	• *Corynebacterium* was the most abundant genera in the penile glans (before and after partnered sexual relationship). • Other abundant penile bacteria in the baseline penile specimen included *Prevotella, Finegoldia, Mycobacterium, Ralstonia*, and *Negativicoccus*.	Carda-Diéguez et al. (70)
To investigate the impact of dual-partner antibiotic treatment of symptomatic BV on the vaginal and penile microbiota	21 HIV-negative men (including 4 circumcised) with female partners were recruited, treated twice daily for 7 days with oral metronidazole 400 mg and/or 2% clindamycin, and followed for 3 weeks post-treatment	• Coronal sulcus and glans • V3–V4 hypervariable region (Illumina MiSeq)	Australian	• Dual-partner treatment of BV had immediate and prolonged effect on cervicovaginal microbiota composition. • Diversity of the cervicovaginal microbiota and prevalence and relative abundance of BV-associated bacteria significantly reduced following treatment. • In men, the effect was immediate, with significant reduction of BV-associated bacteria, including HIV high-risk anaerobes (25). However, the beneficial effect had waned at day 28. • There was by recolonization of the penile microbiota with BV-associated bacteria.	• *Corynebacterium* and BV-associated bacteria (specifically *Finegoldia, Peptoniphilus*, and *Prevotella*) were highly prevalent and abundant in baseline cutaneous penile specimens.	Plummer et al. (71)
To assess the association between penile anaerobic bacteria, cytokines, and HIV acquisition in a case-control study	182 uncircumcised men (46 who became HIV-infected (cases) and 136 who remained uninfected (controls) after 2-years follow-up)	• Coronal sulcus • V3–V4 hypervariable region (Illumina MiSeq)	Ugandan	• Increased absolute abundance of anaerobic *Prevotella, Dialister, Finegoldia*, and *Peptoniphilus* was associated with acquisition of HIV. • Increased absolute abundance of these anaerobes was also correlated with increased levels of chemoattractant cytokine cytokines, particularly IL-8, which can induce an inflammatory response that recruits HIV target cells to the foreskin.	• Not provided.	Liu et al. (25)
To compare the subpreputial microbiota after PrePex device placement to that of uncircumcised men	147 men (including 2 men who underwent a 1-week PrePex device placement and 145 uncircumcised men)	• Coronal sulcus • V3–V6 hypervariable region (454 FLX)	Ugandan	• PrePex users had significantly higher absolute abundance of all penile bacteria compared to uncircumcised men, mainly due to increased absolute abundances of specific anaerobic bacteria. • PrePex users had higher absolute abundance of anaerobic bacteria, mainly *Porphyromonas, Anaerococcus, Peptoniphilus*, and *Campylobacter ureolyticus* compared to uncircumcised controls.	• Anaerobic bacteria (*Peptoniphilus, Anaerococcus, Prevotella, Porphyromonas*, and *Finegoldia*) were common in both PrePex users and uncircumcised men.	Liu et al. (42)
To determine the penile skin, male urethral, and vaginal microbiota of heterosexual couples with and without BV	93 HIV-negative men (including 56 circumcised), matched with their female sexual partners who were either negative or positive for BV	• Glans, coronal sulcus, and shaft • V4–V6 hypervariable region (454 FLX)	USA	• Penile skin and urethral microbiota of males with BV-positive female partners were significantly more similar to their partners' cervicovaginal microbiota compared to the cervicovaginal microbiota of non-partner women with BV. • Penile skin diversities of males with BV-positive partners were significantly higher than that of males with partners without BV.	• *Corynebacterium, Prevotella, Peptoniphilus*, and *Anaerococcus* were the most predominant bacteria in the penile skin microbiota. • *Corynebacterium, Staphylococcus*, and *Gardnerella* were highly abundant in circumcised men. • *Prevotella, Porphyromonas, Anaerococcus, Streptococcus*, and *Finegoldia* were highly abundant in uncircumcised men.	Zozaya et al. (41)
To assess the relationship between penile microbiota of uncircumcised men and Nugent-BV in female partners	165 uncircumcised HIV-negative men who were enrolled in a randomized trial of medical male circumcision for HIV prevention while their female partners were enrolled into a parallel study	• Coronal sulcus • V3–V6 hypervariable region (454 FLX)	Ugandan	• Coronal sulci microbiota clustered into 7 CSTs (designated 1-7 according to increasing bacterial density). Men in CST4-7 had higher bacterial densities compared to men in CST1-3. • Men with multiple extramarital partners and female partners with Nugent-BV were more likely to be in CST4-7 than their counterparts.	• CST1-3 had higher prevalence and relative abundances of *Corynebacterium, L. vaginalis*, and *Staphylococcus* than CST4-7. • CST4-7 had increased prevalence and relative abundances of BV-associated bacteria such as *Porphyromonas*.	Liu et al. (72)
To compare the coronal sulcus microbiota of uncircumcised and circumcised men at enrolment and 1-year follow-up	156 HIV-negative men uncircumcised men [randomized to either immediate circumcision (intervention group) or circumcision delayed for 24 months (control group)]	• Coronal sulcus • V3–V6 hypervariable region (454 FLX)	Ugandan	• At baseline, the prevalence of coronal sulcus bacteria was similar between intervention and control groups. • Male circumcision significantly reduced the bacterial load by reducing both the prevalence and absolute abundance of several anaerobic coronal sulcus bacteria. • Aerobic *Kocuria* and facultative anaerobic *Facklamia* became prevalent after circumcision.	• *Prevotellaceae, Clostridiales Family XI*, unclassified *Clostridiales*, and *Corynebacteriaceae* were the most abundant coronal sulcus bacterial families at baseline. • The most predominant baseline bacteria were: *Prevotella* spp., unclassified *Clostridiales*, and *Corynebacterium* spp. • Other predominant bacteria: *Peptoniphilus, Anaerococcus, Finegoldia, Murdochiella, Porphyromonas*, and *Lactobacillus*.	Liu et al. (57)
To characterize the microbiota of the coronal sulcus and determine the microbiota stability over three consecutive months	18 adolescents (including 5 circumcised) provided baseline specimens and followed at monthly intervals over a 3-months period	• Coronal sulcus • Full-length • V1–V3, V3–V5, and V6–V9 hypervariable regions (454 FLX)	USA	• Coronal sulci microbiota and *Staphylococcus, Mobiluncus, Prevotella, Dialister*, and *Anaerococcus* were relatively stable over time. • BV-associated taxa such as *Prevotella, Atopobium, Mobiluncus, Megasphaera*, and *Gemella* were detected in coronal microbiota of both sexually-experienced and inexperienced participants. *Pseudomonas* in coronal sulcus microbiota was less abundant than previously reported (40).	• *Corynebacterium, Staphylococcus*, and *Anaerococcus* were the most abundant taxa in coronal sulci microbiota. • *Staphylococcus* was highly abundant in circumcised men while *Porphyromonas* and *Prevotella* were abundant in uncircumcised men.	Nelson et al. (73)
To assess the impact of circumcision on the penile microbiota	12 HIV-negative men were randomized to either immediate circumcision (intervention) or to circumcision delayed for 24 months (controls) and were followed at 6, 12, and 24 months	• Coronal sulcus • V3–V4 hypervariable region (454 FLX)	Uganda	• Anaerobic families, *Clostridiales Family XI* and *Prevotellaceae*, were significantly abundant in pre-circumcision coronal sulci samples. • Coronal sulci microbiota was less heterogeneous after circumcision. • After circumcision bacterial families consisting of predominantly anaerobic genera were significantly decreased whereas predominantly facultative anaerobic genera increased. • Aerobic/facultative anaerobic family *Corynebacteriaceae* and a facultative anaerobic *Staphylococcaceae* were significantly abundant in post-circumcision coronal sulci samples. • *Pseudomonadaceae* and *Oxalobacteraceae* were positively correlated, but negatively correlated with *Clostridiales Family XI, Prevotellaceae, Corynebacteriaceae*, and *Staphylococcaceae*.	• Of the 42 bacterial families identified, *Pseudomonadaceae* and *Oxalobacteraceae* were the most abundant, regardless of the circumcision status.	Price et al. (40)

*NGS, next generation sequencing; HPV, human papillomavirus; HR-HPV, high-risk human papillomavirus; CST, community state type; H_2_O_2_, hydrogen peroxide; BV, bacterial vaginosis; HIV, human immunodeficiency virus; IL, interleukin*.

#### Bacterial Changes in Penile Microbiota Following Male Circumcision (Evidence From Longitudinal Studies)

The human penis is inhabited by diverse bacterial families, including *Corynebacteriaceae, Pseudomonadaceae*, and *Oxalobacteraceae* (40, 53, 57). The abundances of these families was shown to be positively or negatively correlated with each other (40, 53). Positive correlation is an indication of cooperative interaction through metabolic resource overlap (74), whereas negative correlation might indicate competition for resources (74, 75) and subniche differentiation (75). The penile bacteria have different aerotolerance profiles (aerobic, anaerobic, facultative anaerobic, and microaerophilic) and therefore are likely to be affected by circumcision (40, 53, 57). Aerobes require oxygen for respiration and growth, whereas anaerobes do not (76). On the other hand, facultative anaerobes can survive in the presence or absence of oxygen, although their growth activity in the oxygen-free environment is usually slower (76). Microaerophiles grow in the presence of oxygen but are sensitive to high oxygen concentrations (77).

A study by Price et al. (40), which examined the effect of circumcision on the coronal sulcus microbiota in 12 HIV-negative Ugandan men of the age 15–49 years, found that circumcision was significantly associated with decreased and increased abundances of anaerobic and facultative anaerobic bacteria, respectively, at 1-year post-circumcision. Specifically, circumcision reduced anaerobic families, including *Clostridiales Family XI* and *Prevotellaceae*, whereas facultative anaerobic families, specifically, *Corynebacteriaceae* and *Staphylococcaceae*, increased (40). In spite of the small study sample size, these findings were confirmed in a subsequent study on a similar Ugandan cohort (77 uncircumcised controls and 79 circumcised intervention group) by Liu et al. (57), hence these results may be generalizable. Here, circumcision considerably reduced the prevalence and density of several anaerobic bacteria (e.g., *Prevotella* spp., *Finegoldia* spp., and *Porphyromonas* spp.), bacterial shifts that were evident year one post-circumcision. Even though the overall prevalence of anaerobic bacteria, including those in *Clostridiales Family XI* reduced post-circumcision, the prevalence of certain anaerobes, particularly *Sneathia* spp., *Atopobium* spp., and *Megasphaera* spp., did not statistically differ before and after circumcision (57). Consistent with observations by Price et al. (40), there was a considerable increase in proportional abundances of *Corynebacterium* and *Staphylococcus* (both facultative anaerobes). Although Liu et al. (57) noted that aerobic bacteria increased post-circumcision, these changes were not significant. This corroborated the finding by Price et al. (40) that suggests that circumcision does not have a significant impact on aerobic bacteria. In addition, both studies agreed that male circumcision results in a more homogenous coronal sulcus microbiota with reduced bacterial diversity (40, 57).

#### Penile Microbiota of Uncircumcised vs. Circumcised Men

Studies that have examined penile microbiota using cohorts of uncircumcised and circumcised men (25, 41, 53, 70–73) found notable differences in the penile microbiota of these two groups of men—observations that overlap with those of longitudinal studies (40, 57). For instance, higher relative abundances of *Porphyromonas* (6.4 vs. 0.3%) and *Prevotella* (12.9 vs. 0%) and lower relative abundance of *Staphylococcus* (5.5 vs. 26.6%) were found in the coronal sulci of 5 uncircumcised adolescent men compared to 12 circumcised adolescent men (73). Recent studies with larger cohorts (93–182 men) confirmed these results (25, 41). *Prevotella* and *Staphylococcus* are among the coronal sulci bacteria that were found to be relatively stable over time (70, 73). *Staphylococcus* is a predominant and stable skin commensal (2). Other predominant bacteria include *Corynebacterium, Finegoldia, Gardnerella, Anaerococcus*, and unclassified taxa, which appear to vary in prevalence and/or relative abundance by the penile site sampled and/or circumcision status (41, 53, 71, 73). While *Corynebacterium*, a Gram-negative and predominant skin colonizer (2), continues to be reported as a common bacterium in the penile microbiota (25, 40, 41, 53, 57, 70–73), its role remains under-appreciated since it is a fastidious bacterium that is difficult to culture (2).

The recent decade has witnessed new studies with interest to unravel the diversity of penile microbiota of uncircumcised and circumcised men. One such study used coronal sulcus specimens collected from 18 adolescents in Indiana, U.S. (73). Diversity analysis using weighted UniFrac distances found that coronal sulci microbiota clearly differentiated according to circumcision status (73). This was in line with studies assessing changes in the composition and diversity of the coronal sulci microbiota following circumcision (40, 57). However, a study that assessed the microbiota in penile skin (glans, coronal sulcus, and penile shaft) of 93 heterosexual U.S. adult men from New Orleans found no significant difference in the weighted UniFrac distances of penile microbiota of circumcised and uncircumcised men (41). Differences in study design, study populations, and study methodologies may at least partly account for the inconsistency in data. Such is not limited to differences in (i) geographical locations (Indianapolis vs. New Orleans), ii) age and sexual behavior of participants (sexually-experienced and inexperienced adolescents aged 14–17 years vs. sexually active adults aged ≥18 years), (iii) prevalence of circumcised men [70.6% [12/17] vs. 60.2% [56/93]], (iv) sampling sites (either coronal sulcus alone or coronal sulcus plus glans and penile shaft), (v) choice of the 16S rRNA region (V1–V3, V3–V5, and V6–V9 vs. V4–V6), and confounders. All these factors have potential to affect the diversity of microbiota (2, 40, 78–80). It is thought that besides male circumcision, participant behavior may also influence the coronal sulci microbiota (57). Since it has been recommended for newly circumcised men to abstain from sex for 6 weeks to allow their wounds to fully heal (66), it is very likely they have reduced exposure to vaginal bacteria compared to uncircumcised men or circumcised men with fully healed penises. Frequent partnered sexual activity may therefore mask the impact of circumcision on the diversity of the penile microbiota (41). This could be due to period colonization of the glans and coronal sulci by vaginal bacteria (70, 81). While the two studies on adolescents and adults men from the U.S. used similar sequencing technology (454 FLX), it is also possible that the difference in the choice of the hypervariable region of the 16S rRNA gene led to the inconsistencies in the diversity results. Even under the same sequencing technology such as 454 FLX, various configurations of the hypervariable region of the 16S rRNA gene may yield differing results (78), thus affecting beta diversity estimations (79).

It is worthwhile underlining that the prevalence and abundances of certain penile bacteria have been correlated with bacterial diversity of the penile microbiota. A study that sought to characterize the composition and density of penile microbiota using coronal sulci swabs from 165 uncircumcised HIV-negative Ugandan men found that the bacterial communities clustered into seven distinct community state types (CSTs, designated CST1 to 7 according to increasing bacterial densities) (72). These CSTs could further be clustered into two groups, CST1-3 (prevalence: 61.2%) and CST4-7 (72). CST1-3 had lower total bacterial density, and higher prevalence and relative abundances of *Corynebacterium* and *Staphylococcus* than CST4-7 (72). In contrast, CST4-7 had higher relative abundances of unclassified *Clostridiales*, unclassified *Clostridiales Family XI*, unclassified phyla, and bacterial vaginosis (BV)-associated bacteria like *Prevotella* and *Porphyromonas* (72). Recently, we described 6 CSTs of the penile microbiota using glans, shaft, and foreskin swabs collected from 238 South African men, mostly circumcised (94.3%) (53). CST-1 (prevalence: 53.4%) and CST-6 (2.5%) were dominated by *Corynebacterium* and *Lactobacillus*, respectively. CSTs 2-5 (44.1%) were more diverse than CST-1 and CST-6 and were associated with higher relative abundances several bacteria (e.g., *Pseudomonas, Hallella, Sutterella, Olsenella*, and *Kocuria*), including BV-associated bacteria (53), similar to CST4-7 in the Ugandan study (72). It should, however, be noted that the Ugandan and our South African cross-sectional studies sampled different penile microenvironments of men with different sociodemographic characteristics, sexual behaviors, and clinical history; warranting caution in interpreting these results in the context of other cross-sectional samples and populations.

Male circumcision is also believed to impact the predominance of pathogenic and potentially pathogenic bacteria in the penis. A study assessing the bacteria in the coronal sulci of 315 circumcised and uncircumcised South Indian men observed that opportunistic pathogens were independently associated with uncircumcised status (3). Specifically, Gram-positive (e.g., *Staphylococcus aureus* and *Enterococcus* spp.), Gram-negative (e.g., *E. coli, Pseudomonas aeruginosa*, and *Klebsiella* spp.), and any opportunistic pathogen (e.g., *Clostridium* spp.) were over two to three times more likely to occur in coronal sulci of uncircumcised than of circumcised men (3).

### Temporal Stability and Individuality of the Penile Microbiota

So far, only two studies have assessed the stability of penile microbiota, specifically, that of the glans (70) and coronal sulcus (73). The stability of other penile microenvironments is still unknown. In a study that examined the stability of coronal sulcus and urine microbiota of 18 healthy adolescents (4 Latino, 7 Black, and 7 White American aged 14–17 years) over three consecutive months, it was observed that the coronal sulcus microbiota was significantly more stable (Sørenson similarity coefficient: 0.60) than urine microbiota (0.52) (73). Comparison of intrapersonal and interpersonal similarity of coronal sulcus microbiota using weighted and unweighted UniFrac distances, the Sørenson similarity index and Spearman correlation coefficient demonstrated that coronal sulcus specimens from the same individual were significantly more similar than specimens from other individuals (73). Individuality in microbiota, of skin for instance, has been published (2). Whereas, *Staphylococcus, Mobiluncus, Prevotella, Dialister*, and *Anaerococcus* were found to be stable members of the coronal sulcus microbiota as measured by Lin's concordance correlation coefficients (mean values of ≥0.5) (73), bacterial taxa such as *Veillonella, Delftia*, and *Streptococcus* were not (mean values of between 0.00 and 0.25) (73). Unstable coronal sulcus bacterial taxa might be synonymous with transient colonizers of the coronal sulcus.

A recent case-control study shed further light on the stability of penile microbiota. This study sought to determine the impact of oral and vaginal sex over the oral and genital microbiota of a 34-years old uncircumcised Spaniard and her 32-years old female sexual partner who reported recurrent vaginal syndromes and gingivitis after sexual intercourse (70). Two time point penile specimens revealed that in absence of sexual activity, the glans microbiota was relatively stable over a short period of time, with minor changes (70). *Corynebacterium* had infinitesimal changes between baseline and follow-up specimens (70), an indication that it could be a core species in the penile microbiota. Condomless sexual intercourse was observed to impinge the glans microbiota, manifested by ~2–10-fold increase in the relative abundances of *Corynebacterium, Lactobacillus, Pelomonas, Ralstonia*, and *Mycobacterium* concomitant with about 3–142-fold decrease in the relative abundances of *Dialister, Megasphaera, Shuttleworthia, Atopobium*, and *Prevotella* (70). It was further observed that one-day treatment of the penis with hydrogen peroxide (H_2_O_2_) in order to alleviate symptoms of sex-derived pathology in the female sexual partner did not affect the composition of the glans microbiota (70). However, after 15 days of intermittent H_2_O_2_ treatment, penile *Prevotella* was eliminated whereas the abundance of *Klebsiella* increased (49%) (70).

Even though the two studies on stability of the glans and coronal sulcus microbiota over a short period of time have laid a foundation for future-related microbiota research, the generalizability of these study findings to other populations are limited given their extremely small samples, different participant ethnicities/races, and different sampling penile sites. Nevertheless, the two studies did not explore the stability of the penile microbiota, including their transitional probabilities, as a function of host and other environmental factors besides recent sexual activity; yet we know that host and environment factors such as age, sex, skin topographical location, clothing choice, and antibiotic use may affect the skin microbiota landscape (2), including that of the penile skin (4, 57, 71). Therefore, one area for future research is to utilize time-series analyses to investigate the long-term stability of the foreskin, glans, coronal sulcus, and shaft microbiota as a function of host and environmental factors. Taxonomic and functional information about resilient and persistent penile commensals may help us elucidate the main drivers of community composition and diversity and bacteria that play significant roles in men's penile health and disease.

### Urine and Urethral Microbiota as a Potential Reservoir for Colonization of the Penile Microbiota

Whereas, limited literature shows that the distributions of bacteria in paired urine-urethral swab samples from the same individual are highly concordant, irrespective of the STI (*Neisseria gonorrhoeae, Chlamydia trachomatis*, and *Trichomonas vaginalis*) status of the subjects, (82), it remains to be determined if the urine/urethral microbiota impacts the penile microbiota, especially the foreskin or glans (of an uncircumcised penis). Of the few available studies that characterized the urethral and/or urine microbiota (73, 82–85), only one has examined the microbiota of both the coronal sulcus and urethra (73). A few caveats of this study comprise, its small sample size (18), inclusion of narrow age of participants (14–17 years), and lack of data on urogenital incontinences and diseases, which limits its generalizability to the wider male population. In this particular study (73), *Corynebacterium* and *Staphylococcus* were the most abundant bacteria in coronal sulcus, while *Streptococcus* and *Lactobacillus* were the most abundant bacteria in distal urethra. In spite of the corona sulci and urine/urethral microbiota being distinct (73), some of the common urine/urethral bacterial taxa, e.g., *Lactobacillus, Staphylococcus, Streptococcus*, and *Corynebacterium* (73, 84, 85), were also found to be abundant in penile (glans, coronal sulcus, penile shaft) microbiota (40, 57, 72). Major urine bacterial taxa such as *Veillonella* and *Streptococcus* have been found not to be stable members of the coronal sulcus microbiota, thus suggesting that these taxa periodically inhabit the coronal sulcus (73). Some of the urethral taxa are assumed to originate from the urethral meatus or coronal sulcus (73). Asymptomatic STIs (*C. trachomatis* and *N. gonorrhoeae*) have been associated with urine microbiota dominated by fastidious, anaerobic and uncultured bacteria such as *Prevotella* spp. and *Sneathia* spp. (84). Since the foreskin (including the acroposthion and frenar/ridged band) and the glans are in close proximity to the urethral meatus, we speculate that disturbances of the distal urethral microbiota by urogenital infections may also affect the microbiota of these adjacent niches. This is because of the assumption that the urine/urethral microbiota may serve as reservoir for colonization of the foreskin, glans, and coronal sulcus microenvironments.

The data presented herein suggests a correspondence between the penile and urine/urethral microbiota. In uncircumcised men, urine may be an important source of bacteria that colonize the glans, coronal sulcus, and preputial sac. This hypothesis can be addressed by longitudinally examining the penile and urine/urethral microbiota of uncircumcised men (with retractile and non-retractile prepuces) and circumcised men or men undergoing circumcision.

### Potential Role of the Penile Microbiota in HIV Acquisition

To date, there are only two molecular studies—a retrospective cross-sectional study from our research group (53) and a case-control study by Liu et al. (25)—suggesting that the penile microbiota may be a risk factor for HIV infection in men.

In our study, we assessed the association of prevalent HIV infection with glans, shaft, and foreskin microbiota of 150 HIV-seronegative and 88 HIV-seropositive heterosexual South African men (53). We observed that the relationship between HIV infection and the alpha diversity (richness, evenness, abundance, and taxon diversity) of the penile microbiota was of borderline statistical significance (*p* < 0.05) (53). The lack of significant association between HIV and penile microbiota or CSTs could be somewhat attributed to recency in age of HIV infection, long-term antiretroviral therapy (ART), or immune reconstitution. This hypothesis is based on the observation that HIV reduces semen microbiota diversity, which is restored after long-term ART, possibly through immune reconstitution (86). We further observed that among the bacterial taxa that were differentially abundant between men with and without HIV infection (e.g., *Staphylococcus, Strenotrophominas, Propionibacterium*, and *Nosocomiicoccus*), it was only an unclassified bacterium in the order *Actinomycetales* that was associated with HIV infection after adjustment for multiple comparisons (53). The association between HIV infection and the relative abundances of certain penile bacteria could mean that such bacteria either increase the risk of HIV infection or occur as a consequence of HIV infection. Thus, further studies would be needed to examine causation and impact of HIV infection on penile microbiota.

In the case-control study, coronal sulcus swabs were collected from 182 uncircumcised heterosexual Ugandan men (25) who had participated in a 2-years RCT of MMC (8). Of these men, approximately 25% (45) were HIV-infected (cases) (25). Since previous investigations postulated that changes in penile microbiota, manifested by reduction of anaerobic bacteria following circumcision, may play a mechanistic role in decreased HIV acquisition (40, 57), the study examined the association between the absolute abundances of selected anaerobic bacterial genera and risk of HIV seroconversion. The selected bacteria had previously been found to significantly reduce post-circumcision (57). Besides observing that these genera constituted, on average, 62% of the total penile bacterial load in the study participants, the aforementioned case-control study found that the 10-fold increased absolute abundances of *Finegoldia, Peptoniphilus, Prevotella*, and *Dialister* on coronal sulci was associated with 54–63% increased risk of HIV seroconversion (25). Our study indirectly echoed part of these observations since we noted a trend toward increased HIV prevalence in men with diverse penile microbiota having low abundance of *Corynebacterium* and dominated by abundances of BV-associated bacteria, specifically *Prevotella* (53). In women, BV-associated bacteria such as *Prevotella* spp. have been associated with increased risk of acquiring HIV (87). There are reports suggesting that BV-associated bacteria, including the penile anaerobes, can be transmitted heterosexually (41, 72). Thus, the literature described here provides hints that some penile anaerobic bacteria may be sexually transmissible risk factors for HIV. The reduction in penile anaerobes may partly account for the reduced risk of heterosexually acquired HIV infection in men following circumcision (57).

The case-control study also demonstrated correlations between the absolute abundances of penile anaerobes (such as *Prevotella, Dialister*, and *Peptostreptococcus*) and elevated levels of chemokines, including IL-8 (25). A case-control study testing the association between proinflammatory penile cytokines and risk of HIV acquisition in 180 Ugandan men (60 cases and 120 controls) showed that detectable levels of IL-8 in the coronal sulcus correlated with both increased density of preputial HIV target cells (including the highly susceptible CD4 T cells subsets) and HIV acquisition (88). IL-8 is one of the chemotactic (HIV target cell-recruiting) cytokines in the cervicovaginal milieu whose elevated level was associated with increased risk of HIV acquisition in women (89). Although no similar studies were performed on men, levels of IL-8 were found to gradually decline following male circumcision (88). Apart from IL-8, the inner foreskin epithelium can secrete high levels of other inflammatory cytokines, such as GM-CSF, IFN-γ, IP-10, and RANTES (33). Therefore, strategies aimed at modifying the penile microbiota and chemotactic cytokines may contribute to reduced risk of HIV transmission.

Uncircumcised males often have foreskins that can retract (36, 68, 69), thereby exposing the squamous mucosa of the penis and coronal sulcus (29, 30). While there are reports that the inner foreskin keratin layers (or stratum corneum) are considerably thinner than the outer foreskin (33), other investigations have found no differences (31, 32). Difference in susceptibility to HIV/STIs between the outer and inner foreskin is likely to be due to differences in target cells and permissiveness of the epithelium layers to HIV/STIs. The mucosal epithelial of the penis is thought to be more susceptible to HIV compared to other keratinized epithelia. Since a larger surface area of the foreskin has been identified as a potential risk factor for HIV in men (38), it is tempting to think that a larger surface area of the foreskin may be enriched in HIV target cells and offer more anoxic microenvironment, which in turn supports penile colonization with a diverse array of anaerobes. The penile anaerobes may cause local inflammation, which is conducive to HIV (20). Moreover, penile ulcerative or inflammatory lesions caused by STIs may provide additional routes for HIV transmission (22). Studies on women have associated genital inflammation with increased risk of HIV infection (89). These heightened genital inflammatory responses are elicited by specific cervicovaginal bacteria (87, 90). Reportedly, compared to circumcised men, uncircumcised men have higher loads of anaerobes (57) that presumably may create proinflammatory milieus, potentially activating the Langerhans cells to present the HIV to macrophages, dendritic cells, and T cells immune cells (40, 88). STIs such as *N. gonorrhoeae* may also enhance HIV transmission by recruiting and activating HIV target cells at the site of infection (27). A comparative immunohistological investigation of human and non-human primate oral, cervicovaginal, foreskin, urethral, and rectal epithelia for potential HIV receptors found Langerhans cells in the foreskin epithelium but not the urethral epithelium (91), suggesting that, unlike the foreskin epithelium, the urethral epithelium might not be a common site of entry for HIV.

Compared to the outer foreskin, the underlying foreskin mucosal tissues are highly lined with HIV target cells (macrophages and CD4^+^ T cell subsets such as Th17 cells and those expressing the HIV co-receptor CCR5 and α4β7 receptors) (24, 31, 33, 43, 91) and lymphoid aggregates (T cells, CD209^+^ dendritic cells, and CD68^+^ macrophages) (24), potentially making such tissues susceptible to HIV. A foreskin explant culture model observed that HIV-1 replication (as manifested by the accumulation of p24 antigen) was slightly higher in the supernatant inner foreskin-derived explants than outer foreskin-derived explants. Although this result suggests that HIV-1 replication may be more efficient in the inner foreskin relative to the outer foreskin tissue, caution must be taken when drawing conclusions from this result since the difference in HIV-1 replication between the two foreskin sites was not significant (24). In another study that explored possible sites for HIV transmission across the penis (31), HIV was more likely to interact with the inner foreskin or uncircumcised glans than the outer foreskin. Furthermore, CD4^+^ T cells were slightly more in the uncircumcised glans epithelia compared to the shaft epithelia and occurred closer to the epithelial surface, albeit not significantly closer (31). While a total of 12 foreskin and 14 cadaveric penile tissue specimens were included in this study, additional research using more specimens is required prior to drawing firm conclusions regarding differential compartmentalization of HIV-susceptible penile cells. This study further observed that CD4^+^ T cells were closer to the surface of penile shaft tissues of uncircumcised donors compared to circumcised donors (31). In circumcised donors, CD4^+^ T cells were closer to the surface of the glans compared to the shaft tissue (31). Generalizability of this data is also uncertain since experiments on cadaveric tissues may not necessarily be reproduced in non-cadaveric tissues. However, from these findings, it can be argued that, the glans, besides the foreskin, may be permissive to HIV infection through CD4^+^ T cells and that the glans could be one of the key sites for HIV infection in both uncircumcised and circumcised men. A relatively recent murine model illustrated that activated mucosal CD4^+^ T cells increased in the vagina of germ-free mice intravaginally administered with *Prevotella bivia* (87). Similarly, there could be interactions between penile anaerobic bacteria and HIV target cells in the foreskin and uncircumcised glans.

Together, the investigations discussed here point to the involvement of the penile microbiota (particularly of uncircumcised men), coupled with immune activation responses, in the acquisition and transmission of HIV. We now know that the subpreputial space is a home to a pool of anaerobic bacteria (40, 57) and that the subpreputial mucosal immune milieu is proinflammatory in nature (43). The penile bacteria may drive genital immune activation, thereby increasing susceptibility to HIV infection (20). There is a possible link between HIV reduction and changes in penile microbiota. This reasoning comes from the evidence that male circumcision reduces the risk of HIV acquisition (6–8) and the diversity and density of anaerobic bacteria (40, 57). Although the mechanism is likely more complex than presented herein, changes in the immunobiology of the penis are one of the plausible explanations of how circumcision reduces the risk of HIV infection.

### Potential Role of the Penile Microbiota in HPV Infection

Since male circumcision has been associated with changes in penile microbiota (40, 57) and reduced risk of HPV (13–15, 18, 55, 56), including high-risk (HR) (18, 19) and multiple HPV infections (18), it is therefore reasonable to imagine that there is an association between penile microbiota and HPV infection. To the best of our knowledge, our molecular study on 238 South African men is the first and only one to examine the association between the penile microbiota and HPV infection (53). In this study, 54.6 and 42.9% of the men were positive for HPV and HR-HPV infections, respectively, (53). Men with *Corynebacterium*-dominated penile microbiota were less likely to have HR-HPV compared to men with pooled non-*Corynebacterium*-dominated penile microbiota, including the ones dominated with BV-associated bacteria or *Lactobacillus* (53). Men with diverse penile microbiota, specifically dominated by *Prevotella, Clostridiales*, and *Porphyromonas* and a lower relative abundance of *Corynebacterium* were more likely to have HPV or HR-HPV infections than men with *Corynebacterium*-dominated penile microbiota (53). We used a machine learning approach to identify the bacterial taxa that were differentially abundant in men with vs. without HPV and HR-HPV infections. We noted that higher relative abundances of BV-associated bacteria (*Prevotella, Peptinophilus*, and *Dialister*) and lower relative abundance of *Corynebacterium* were distinctively associated with HR-HPV infections (53). It seems, therefore, that there might be a link between *Corynebacterium* and protection against penile HPV infection.

In an attempt to investigate the impact of HPV infection with or without HIV co-infection on penile microbiota, we used different diversity indices. We found that men with viral co-infections had significantly higher alpha diversity than HIV-negative men with and without HPV infection (53). Additionally, HR-HPV-positive men with HIV infections had significantly higher alpha diversity than HR-HPV-positive men without HIV infection (53). In women, high diversity cervicovaginal microbiota has been associated with HPV infection (92). Though the data on the association between penile microbiota and HPV did not support an altered penile microbiota in the causation of HPV or vice-versa, the data suggest that the impact of HPV or HR-HPV infection on penile microbiota diversity may be enhanced with HIV co-infection. It is well established that there is a complex interplay between HIV and HPV infection. Whereas, HPV infection increases the risk of HIV infection, HIV increases the risk of acquisition, persistence, and reduces the clearance of HPV infection (93). As a consequence, this may alter microbiota, including that of the penis. This HPV-associated microbiota changes, together with whether the changes in penile microbiota predispose men to HPV infection remains to be determined. It is also possible that the penile HPV was not an established infection but deposition following recent sexual activity. Therefore, future longitudinal studies will be needed to validate these claims and determine the interplay between temporal changes in penile microbiota and natural history of HPV infection.

Inasmuch as there are no studies linking host immune responses with penile microbiota and penile HPV infection, we think that the penile microbiota may modulate host immunity to HPV infection. This hypothesis is based on cervicovaginal microbiota study that not only associated diverse microbiota with HPV infection, but with elevated levels of chemokines IP-10 and MIG (92). HPV remission was associated with increased Langerhans cells (92). This clearance could be because the Langerhans cells aid in antigen recognition, processing, and presentation to macrophages and lymphocytes. In our penile microbiota study (53), HPV-positive men with diverse microbiota could be having higher local chemokines compared to men with *Corynebacterium*-dominated microbiota. There could also be an interaction between *Corynebacterium* and Langerhans cells that facilities clearance of HPV infection. Differences in site-specific genital HPV infection and clearance in uncircumcised and circumcised men (17, 18, 56) could be due to differences the distribution penile chemokines and Langerhans cells. Future studies should thus focus on investigating the penile immunobiology in penile health and disease.

### Genital Microbiota Sharing Between Heterosexual Couples

#### Role of Penile Microbiota in Shaping Cervicovaginal Microbiota

BV, the most common vaginal disorder among reproductive-age women, particularly in sub-Saharan Africa (94, 95), is characterized by a substantial depletion and/or displacement of *Lactobacillus* spp., which are supplanted by an overgrowth of facultative anaerobic and/or anaerobic bacteria that often include *Gardnerella, Prevotella, Mobiluncus, Mycoplasma*, and *Porphyromonas* (96).

There are several risk factors for BV, including genetics (97, 98), STIs (98, 99), physiological (97, 100), and sociobehavioural factors such as partnered sexual activities (70, 97, 98, 101–103).

BV has an impact on women's health. For example, BV is associated with 60% increased risk of HIV acquisition in HIV incidence studies (relative risk (RR): 1.6 [95% confidence interval (CI) 1.6 1.2–2.1]) (104). A systematic review showed a positive association between BV and uterine cervical HPV infection (odds ratio: 1.4 [95% CI 1.1–1.8]) (105), thus signifying that BV increases the risk of cervical HPV infection.

Lately, there has been considerable discussion regarding the genital microbiota of couples (41, 70, 71, 106) and recognition that BV-associated bacteria may be sexually transmitted between partners (70–72, 106–109). The penis of sexually-experienced and inexperienced men can be a reservoir of BV-associated bacteria (25, 40, 41, 53, 57, 70–73) and other bacterial pathogens (3, 4), which are often reduced by male circumcision (40, 41, 57). The penile bacteria may perhaps influence BV status in the female sexual partner (70, 71). Furthermore, it is thought that persistent or recurrent BV in women could be primarily due to heterosexual exposure to BV-associated bacteria (70, 102, 103, 108). Thus, changes in penile microbiota could explain why male circumcision drastically reduces the risk of BV in female sexual partners (11, 15). Although many studies have been published on the human genital microbiota, the molecular and microbiological studies that definitively demonstrate the sexual exchange of BV-associated bacteria are limited.

Evidence of shared microbiota between partner pairs has been observed in urogenital microbiota (41, 70–72, 106, 107). Studies on this topic together with the role of male partner in shaping the cervicovaginal microbiota are scarce (70, 106, 107, 110). The few available studies have documented heterosexual transmission and concordance of BV-associated bacteria (41, 70, 72, 107). A study characterizing the genital microbiota of heterosexual couples with and without BV observed that the genital microbiota of BV-couples (male partners with female partners having BV) became more similar over time, regardless of the circumcision status of the male partner (41). This extends previous investigations that observed that heterosexual men with penile microbiota dominated by BV-associated bacteria were more likely to have marital sexual partners with BV and that having extramarital female partners was significantly associated with such penile microbiota (72). A recent case report found that condomless penile-vaginal intercourse augmented BV-associated bacteria (specifically *Atopobium* and *Prevotella*) in the vagina and resulted in vaginal dysbiosis (70). These results suggest that sexual intercourse directly influences the genital microbiota.

Condomless receptive vaginal sex may increase the risk for BV acquisition and recurrence (70, 97, 102, 103, 109) because of the possible heterosexual transmission of bacteria, including BV-associated bacteria (41, 70, 72, 110). There are still conflicting reports in the literature about the effects of sexual intercourse on the vaginal microbiota, with studies reporting either a substantial depletion of *Lactobacillus* (70, 106) including *Lactobacillus crispatus* (111) or no effect/loss of lactobacilli (108, 112). Furthermore, while some researchers (112) have observed that only the concentrations of *E. coli* significantly increase after sexual intercourse, others have observed either emergence of BV-associated communities (70, 110) or no bacterial changes apart from gain in colonization with *G. vaginalis* (108). Vaginal colonization with *G. vaginalis* was found to be more common in young women who engaged in sexual activity than in virgins (108). A current longitudinal study on a young Australian cohort showed that although sexual intercourse did not affect the stability of vaginal microbiota, it was associated with increased diversity of *G. vaginalis* clades (113). Of the multiple clades of *G. vaginalis* (GV1, GV2, GV3, and GV4), the clade GV4 was positively associated with incident and prevalent BV (113). The variations in the observed effects of sexual intercourse on the vaginal microbiota could be explained by the heterogeneity of study design and study populations.

A large cohort longitudinal study assessing the risk factors of BV among North American women from different ethnicities aged 18–30 years associated incidental BV with sexual intercourse (with uncircumcised men) and receptive anal sex prior to penile-vaginal sexual intercourse (98). Although there is clinical and epidemiological evidence that sexual exposure increases the risk of BV (70, 101, 102), including its recurrence (103), BV is not recognized as an STI (102, 114); neither is it considered as a sexually enhanced disease (SED) as proposed by Verstraelen et al. (115) a decade ago. A systematic review and meta-analysis (102) reported that having new and multiple male sexual partners increased the risk of BV by 1.6-fold (RR: 95% CI 1.5–1.8), while a history of female sexual partners increased the risk of BV by 2-fold (RR: 95% CI 1.7–2.3). It was further observed that consistent condom use is associated with reduced incident and recurrent BV (102). This suggests that the penile microbiota might have a significant effect on the cervicovaginal microbiota.

#### Impact of Modifying Penile Microbiota on Female Partner's Bacterial Vaginosis Status

Currently, BV remains a microbiological enigma in human reproductive health and presents a treatment challenge due to its complex polymicrobial nature. According to observational evidence based on five out of six RCTs with different methodologies (114), male partner treatment with antibiotics does not have a beneficial effect in reducing the risk of BV in the female sexual partner. However, in contrast to this, a pilot study on the effect of dual partner treatment on BV (i.e., treatment of BV-positive female and her sexual male partner) in 21 Australian couples (71) points to a possible beneficial effect of treatment of the male sexual partner in preventing recurrence of BV in the female partner. This study observed an immediate and prolonged decrease in predominance of BV-associated bacteria, reduction in bacterial diversity, and increased predominance of *Lactobacillus iners* in the cervicovaginal microbiota 3 weeks post-treatment (71). This study also showed that antibiotics for treatment of BV had a short-term effect on the cutaneous penile microbiota, since BV-associated bacteria re-emerged shortly after treatment (71). Most of the cutaneous penile microbiota were recolonized with BV-associated bacteria 3 weeks post-treatment (71). The inability to clear BV-associated bacteria inhabiting the penis could be one of the primary reasons why BV recurs and persists in women. Men with BV-positive female partners have been found to have an ~2-fold increase in bacterial density compared to men with BV-negative partners (72). Lack of beneficial effect in reducing the risk of BV in the female sexual partner after the male partner treatment (as observed in several RCTs), coupled with its short-term beneficial effect in the treated male partner, is one of the primary reasons why BV is not regarded as an STI, despite its similar epidemiological profile (in terms of associations with sexual risk factors) to established STIs (102). Moreover, there is no single bacterial pathogen (not even from the penis) that has been identified to be responsible for the etiology of BV (102).

### Gaps and Challenges in the Current Literature and Future Prospects

Peer-reviewed publications on the human penile microbiota and their potential impact on STIs and cervicovaginal microbiota are still scanty. In spite of the differing bacteriological observations, it is widely accepted that condomless sexual intercourse influences the cervicovaginal microbiota (70, 102, 103, 113) and that the penis is a potential vector for transmission of BV (41, 70–72, 107). However, the exact contribution of the penile microbiota on BV etiology remains unknown. Hence, carefully constructed studies of genital microbiota of heterosexual partners will help in delineating the poorly understood etiology of BV and establish whether it is a SED as proposed (115).

The penile microbiota and its associations with STIs present an interesting area of research. Currently, there are no published reports on the association between penile microbiota and STIs other than HIV and HPV, despite the following:

- High global burden of the other STIs (95).
- Association of male circumcision with reduced risk of STIs (12, 15, 19).

The hypotheses that penile microbiota could impact other STIs ought to be explored, with a view to harness the protective features of the penile microbiota. It would be desirable and informative to investigate the impact of the penile microbiota on the proliferation of pathogenic microbes, including STIs. This could be buttressed by large-scale longitudinal studies examining the temporal dynamics and functional potential of the penile microbiota in relation to the age of an individual, sexual behavior, penile hygiene practices, and so on. At present, the cross-sectional nature of most penile microbiota studies limits any inference of causality and our knowledge of what constitutes a healthy penile microbiota.

Following the association of a larger foreskin size with HIV acquisition (38), researchers should assess whether the foreskin size is related to density of proinflammatory anaerobic bacteria. In addition, the proinflammatory nature of the foreskin should be examined if indeed it is elicited by bacteria or it is an inherent penile feature devoid of bacterial influence. With regard to HPV infection, the immunobiology of the penis should be examined in closer detail in order to unravel the reasons behind the heterogeneity of HPV prevalence and clearance rates in the different penile sites (17, 18, 56).

We are aware that in many regions in sub-Saharan Africa, TMC is common and may not offer the same level of protection against STI/HIV as MMC (6, 10, 15, 45, 47, 63). This is probably due to differences in penile microbiota and immune responses (3, 25, 88). Thus, well-designed studies are needed to investigate how the penile microbiota of traditionally circumcised men compares to those of medically circumcised men and how these microbiota impact STI/HIV.

In the available literature on penile microbiota, there seems to be a growing consensus on the existence of unclassified bacterial taxa inhabiting the penis, especially in the order *Clostridiales* (40, 41, 53, 57, 72). These could either be contaminants or novel bacteria. Therefore, future penile microbiota studies should be conducted using bioinformatics tools such as Divisive Amplicon Denoising Algorithm 2 (DADA2) (116) that can allow accurate classification of sequences to deeper taxonomic ranks and discovery of sequence variations (116). Nonetheless, the biological roles of novel and other poorly studied penile bacteria such as *Stenotrophomonas* (53, 71) and *Murdochiella* (57), to name a few, should be defined. This will hopefully lead to better understanding of the role of penile microbiota in heath and disease.

Lastly, there are a number of notable caveats in the current literature of penile microbiota that have either led to inconsistent findings or potentially limited cross-study comparisons. These include:

- The issue of generalizability, that is, studies may not take account of baseline differences in the penile microbiota by host and environmental factors. Such factors may contribute to variation in microbiota (2, 4). Generalizability of results to other populations may not be applicable especially if studies do not adjust for potential confounders such as smoking, antibiotic use, dermatological conditions, sexual behavior, and other factors that might influence the penile microbiota and be associated with both exposure and outcome.
- Differences in study design and study population. For instance, a study on an uncircumcised Ugandan cohort found that *Pseudomonas* was highly abundant in the coronal sulcus and glans microbiota (40) whereas a study using a similar molecular approach on an Australian cohort (predominantly uncircumcised) found that *Pseudomonas* was less abundant (mean abundance 0.02%) in the coronal sulcus and glans (71). Instead, *Finegoldia* and *Corynebacterium* were highly abundant in baseline penile microbiota (71). There are a number of possible arguments for such varying findings in penile microbiota. Firstly, host genetic background, immune, demographic, and sociobehavioural differences. Secondly, there is likelihood that low-biomass specimens might have been contaminated by high-biomass specimens, thereby skewing microbiota results (117). Thirdly, reliance on self-report on male circumcision status and differences in number of sampling and performance (sensitivity and specificity) of self- and clinician-collected penile swabs could have had an impact on penile microbiota. In some of the penile microbiota studies, participants were requested to provide information on circumcision status (73) and self-collect the penile swab samples (70, 71). A sizeable error of the circumcision status has been observed with self-reported data (48, 67). The penile microbiota has lower bacterial load compared to other microbiota sites and may be particularly low in self-sampling studies. The penile microbiota studies have swabbed the penile microenvironment either once (70), twice (25, 42, 57, 71, 72), or unknown number of times (41, 53, 73), using either dry (53), or premoistened swabs (25, 42, 57, 71, 72). It is likely that the use of dry or premoistened swabs and number of swabbing influences the amount bacteria recovered from the penile microenvironment. Fourthly, and finally, the human skin, and perhaps that of the penis, exhibits niche partitioning (2), of which sampling might be sensitive to.
- Small sample size in some studies, which may have a low statistical power to detect congruence and differences in the composition of penile microbiota, thus impeding meaningful conclusions and cross-study comparisons. Studies involving few participants may not be adequately powered to avoid type I or II error. This limitation argues for future studies to examine larger cohorts.
- Heterogeneity in study methodologies. Penile microbiota studies have relied on different hypervariable regions of the 16S rRNA gene sequenced on either Illumina MiSeq or 454 FLX platforms. This is despite the findings that microbiota profile is dependent on the choice of the 16S rRNA region (73, 78–80) and sequencing technology (78, 80, 118). In the context of penile microbiota, pyrosequenced V1–V3 and V3–V5 datasets do overestimate *Corynebacterium* while V6–V9 dataset underestimates *Prevotella* (73). Even more strikingly, there is evidence that pyrosequenced V1–V3 dataset fails to capture *Gardnerella* (73). Therefore, there is a probability that some of these methodologies might have missed to disclose certain penile bacteria. Thus, an important consideration for future studies is inclusion of appropriate controls and use of more accurate metagenomic approaches like whole-genome sequencing (WGS) methods that have enhanced microbial resolution and allowed detection of putative functional gene composition of microbiota (119).

## Conclusions

In spite of the human penile microbiota being understudied, available literature suggests that the penis is colonized by a vast array of bacteria, including cervicovaginal bacteria. These penile bacteria may be important in the STI epidemiology in men. Overall, the composition and diversity of penile microbiota is impacted by male circumcision. Circumcision significantly reduces the predominance of anaerobic bacteria (40, 57), including those with proinflammatory potential (25), and HIV target cells, consequently lowering vulnerability to STIs, specifically HIV (40, 88). The penile microbiota may play a substantial role in the natural history of HPV infection. There may be need to exploit pre- and probiotics as interventions for preventing HPV infection and promoting its clearance. The findings on effects of male circumcision and dual therapy for BV on penile microbiota (71) suggest that interventions that modulate the penile microbiota could perhaps be used to reduce the risk of STIs acquisition (in both men and women) and prevent BV and its associated consequences. In the case of HIV reduction, Prodger and Kaul (20) suggest that the modality to alter penile microbiota could be used alone or in combination with topical microbicides; but first, generalizability of published findings to other populations needs to be evidenced. At the present time, translation of penile microbiota findings to clinical practice is lacking.

## Author Contributions

HO and TM conceived the idea. HO wrote the first draft of the manuscript. HO, A-LW, JP, and TM reviewed and edited the paper. All authors read and approved the final manuscript.

## Conflict of Interest

The authors declare that the research was conducted in the absence of any commercial or financial relationships that could be construed as a potential conflict of interest.
